# Histological analysis of (antral) follicle density in ovarian cortex tissue attached to stripped endometriomas

**DOI:** 10.1007/s10815-024-03058-0

**Published:** 2024-03-05

**Authors:** Rozemarijn de Koning, Mathijs D. Blikkendaal, Susana M. Chuva de Sousa Lopes, Lotte E. van der Meeren, Hui Cheng, Frank Willem Jansen, Eileen E. L. O. Lashley

**Affiliations:** 1 Department of Gynaecology and Obstetrics, Leiden University Medical Centre, Leiden, The Netherlands; 2 Endometriose Centrum, Haaglanden Medical Centre, Den Haag, The Netherlands; 3 Nederlandse Endometriose Kliniek, Reinier de Graaf Hospital, Delft, The Netherlands; 4 Department of Anatomy and Embryology, Leiden University Medical Centre, Leiden, The Netherlands; 5 Department of Pathology, Leiden University Medical Centre, Leiden, The Netherlands; 6 Department of Pathology, Erasmus Medical Centre, Rotterdam, The Netherlands

**Keywords:** Ovarian endometriosis, Follicle density, In vitro maturation, Endometrioma surgery

## Abstract

**Purpose:**

When resecting endometriomas with the stripping technique, in the majority of cases, a thin line of adjacent ovarian cortex is attached to the endometrioma. In this study, we performed histological analysis to determine (antral) follicle density in the ovarian cortex tissue attached to stripped endometriomas and assessed patient- and surgical characteristics that could affect this.

**Methods:**

Histological slides of previously removed endometriomas were assessed. Follicles in the attached ovarian tissue were classified according to maturation, and follicular density was determined. Immunofluorescent staining of antral follicles in a subset of endometriomas was also performed.

**Results:**

In 90 out of 96 included endometriomas (93.7%), ovarian tissue attached to the cyst wall was observed. One thousand nine hundred forty-four follicles at different maturation stages were identified (3 follicles/mm^3^). Follicle density was negatively associated with age (*p* < 0.001). Antral follicles (< 7-mm diameter) were present in the ovarian tissue attached to 35 endometriomas (36.5%) derived from younger patients compared to endometriomas where none were detected (30 *versus* 35 years, *p* = 0.003). Antral follicle density was 1 follicle/mm^3^. Based on immunofluorescence, healthy antral follicles were identified in two out of four examined endometriomas.

**Conclusions:**

Ovarian tissue attached to stripped endometriomas holds potential as a non-invasive source for antral follicles. In theory, application of IVM could be an interesting alternative FP option in young patients with endometriomas who undergo cystectomy in order to transform the surgical collateral damage to a potential oocyte source. Our results encourage future research with fresh tissue to further assess the quality and potential of these follicles.

**Trial registration:**

Clinical Trials.gov Identifier: B21.055 (METC LDD), date of registration 12–08-2021, retrospectively registered.

**Supplementary Information:**

The online version contains supplementary material available at 10.1007/s10815-024-03058-0.

## Introduction

The presence of endometriomas compromises the ovarian reserve, possibly due to inflammatory reactions and to mechanical induced damage, resulting in depletion of follicles in the surrounding ovarian tissue [1, 2]. Endometriomas are present in 17–44% of women with endometriosis, a population of reproductive-aged women with often a (future) wish to conceive [3]. Removal of these cysts can be considered in patients with large or rapidly growing endometriomas, that suffer from therapy-resistant pain or persistent infertility, or to enable follicle access during the oocyte pick-up procedure in case of in vitro fertilisation (IVF)/intracytoplasmatic sperm injection (ICSI) treatment [4]. Laparoscopic stripping is one of the most widely used techniques to remove endometriomas, which involves surgeons to identify the cleavage plane of the endometrioma pseudocapsule and the ovarian parenchyma, followed by gentle traction to dissect both structures [5]. While this technique can restore anatomy and terminates endometrioma-induced damage, it is known to negatively affect the ovarian reserve, as reflected by significantly decreased anti-Müllerian hormone (AMH) levels and lowered follicle density after surgery, even when performed by experienced surgeons [4, 6]. The decline in AMH is more pronounced in women undergoing bilateral endometrioma cystectomy, which is a known risk factor for premature ovarian insufficiency [4]. It is thought that this damage is induced by injuries to the vascular bed (e.g. due to coagulation or adhesiolysis) and surgery-related edema and inflammation [7]. In addition, multipe histopathologic studies report unintended removal of ovarian tissue (containing follicles in all developmental stages) attached to the endometrioma cysts wall in the majority of cases [8–12].

Fertility preservation (FP) may be considered in this population, particularly given the high proportion of women with endometriosis who may require assisted reproductive technology (ART) to become pregnant [13]. FP options include the preservation of embryos or mature oocytes. However, this requires hormonal stimulation for 2–4 weeks. Due to the expected poor response to gonadotrophin stimulation following endometrioma cystectomy, higher dosages of gonadotrophins and multiple stimulation cycles are often necessary, resulting in increased costs and more risks upon oocyte retrieval [14, 15]. An alternative (experimental) option is ovarian tissue cryopreservation that involves surgical removal of an ovary and subsequent orthotopic reimplantation [16]). This includes two surgical procedures, which could be technically more challenging particularly in cases of pelvic adhesions due to endometriosis [16]. Additionally, ovariectomy further deteriorates ovarian reserve, and the freeze–thaw process results in a decrease in the already low density of viable follicles within the ovary [17]. Hence, there is no optimal method for FP in this population, and it still remains a controversial topic due to a lack of data on efficiency and cost-effectiveness [13].

Unintentionally removed ovarian cortex tissue attached to stripped endometriomas is currently not considered as a potential oocyte source for FP and often is discarded. However, we believe that this tissue may be a potential oocyte source. Previous research shows that this tissue contains follicles of all developmental stages including those follicles with an (small) antrum containing immature cumulus-oocyte-complexes (COCs) [18, 19]. In vitro maturation (IVM) is a cell culture technique that is used to mature COCs at germinal vesicle stage to metaphase II (M2) oocytes. This technique has already been succesfully used for patients who had their ovarian tissue surgically removed for subsequent cryopreservation before undergoing gonadotoxic treatment [20, 21]. However, concerns have been raised regarding the quantity and quality of follicles in the proximity of endometriomas [23, 24].

The objective of this study is to assess the density of (antral) follicles. The primary aim was to quantify and characterise the developmental stages of follicles in ovarian tissue attached to stripped endometriomas. Secondary aims were to identify patient or surgical characteristics affecting follicle density and to look at some viability markers using immunofluorescence.

## Materials and methods

This retrospective cohort study was conducted in two centres, Leiden University Medical Centre (LUMC) and an endometriosis expertise centre within the Haaglanden Medical Centre (HMC). This study was approved by the LDD Medical Research Ethics Committee (B21.055).

### Study population

In the LUMC, the following diagnostic codes were used to search electronic patient records of patients that underwent surgery between 2011 and 2021: “endometriosis”, “ovarian endometriosis” and “ovarian cysts”. In the HMC, all patients that underwent surgery between January 2020 and April 2021 were screened for inclusion. Surgery and pathology reports of all patients were evaluated. Inclusion criteria were women aged 18–50 years, presenting with either unilateral or bilateral ovarian endometriomas, who had undergone laparoscopic cystectomy of an endometrioma as confirmed by histologic analysis. We excluded patients that underwent cystectomy with different pathological diagnosis, other than endometrioma, such as dermoid cyst, cystadenoma, ovarian adenocarcinoma and (mucinous) borderline tumour. Additionally, patients that were mis-coded and underwent adnexal extirpation or coagulation of the cyst rather than cystectomy, were excluded. Baseline characteristics and clinical data were obtained from electronic patient records. Endometrioma cystectomy was performed following the recommendations of the European Society for Gynaecological Endoscopy (ESGE), European Society of Human Reproduction and Embryology (ESHRE) and World Endometriosis Society (WES) working group [5]. The largest perpendicular cross-section of each endometrioma according to the surgical, MRI or ultrasound report, was included as diameter.

### Endometriosis classification

Endometriosis was classified during the surgical procedure by the rASRM score. Classification varied from I, mild endometriosis to IV, severe endometriosis as described previously [25]. Deep endometriosis was classified according to the #Enzian classification using four compartments (A; vagina, rectovaginal space, B; uterosacral ligaments, cardinal ligaments, pelvic sidewall, C; rectum, F; far locations) [26].

### Histochemistry, immunofluorescence and TUNEL assay

**Fig. 1:**
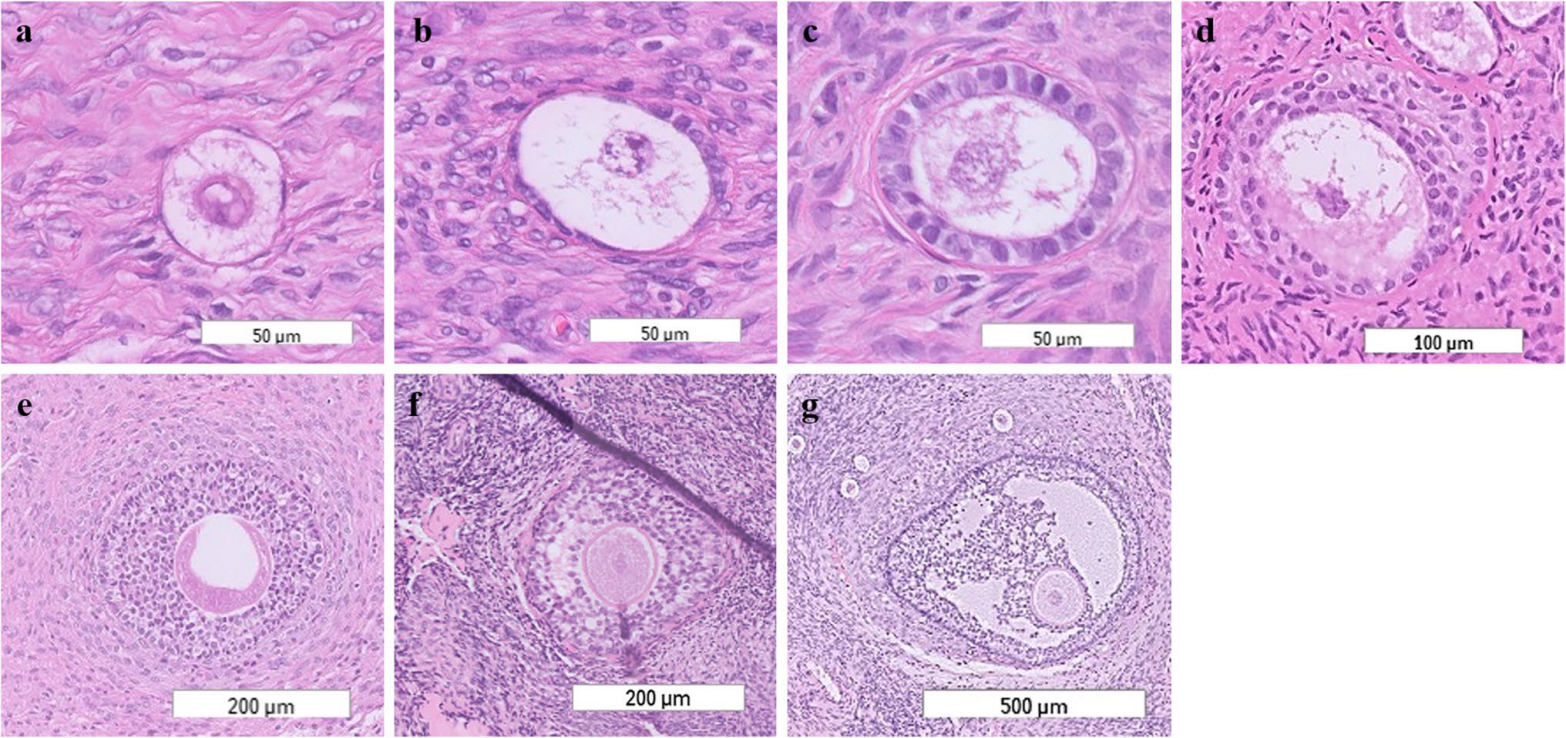
Representative HE images of the used follicle classification system. **a** Follicles were classified as primordial when surrounded by a single layer of squamous granulosa cells. **b** Intermediate primordial follicles were surrounded by a single cell layer that contained both squamous and cuboidal cells. **c** Primary follicles were surrounded with a single layer of cuboidal granulosa cells. **d** Intermediate primary follicles were partially surrounded with a multi-layer and partially with a single layer of cuboidal cells. **e** When the follicle was fully surrounded with a cuboidal cell multilayer, it was staged as late primary. **f** Follicles were classified as small antral follicles when an antrum was observed. **g** If the antrum comprised more than 50% of the follicle, the follicle was staged as antral

Tissue processing was performed according to the local protocol. All slides were digitised (Philips Ultra Fast Scanner), which allowed up to 100 × magnification. The haematoxylin and eosin (HE) slides of previously removed endometriomas were analysed for the presence or absence of ovarian tissue attached to the cysts wall, resulting in the classification of three categories: (1) ovarian tissue with follicles, (2) ovarian tissue without follicles and (3) endometrioma cyst wall and/or scar tissue. Follicle classification was performed by RdK and discussed in case of doubt with a second observer (LvdM or SCdSL). Gougeon’s original follicle classification was modified by adding additional intermediate stages (Fig. 1) [27]. All follicles were counted regardless of the visibility of the oocyte in the available slide(s).The diameter of each follicle was determined by taking the largest perpendicular cross-section between the basement membrane. Follicle surface was determined by delineation of ovarian tissue in each HE slide in ImageJ. Subsequently, the tissue area (mm^2^) was multiplied by the tissue thickness (0.005 mm) to calculate the volume of ovarian tissue (mm^3^) [2]. Follicle density was determined by dividing the number of follicles by the ovarian tissue volume.

Immunofluorescent analysis was performed on ovarian tissue attached to endometriomas containing antral follicles. The sections were deparaffinised and treated with Tris–EDTA buffer (10 mM Tris, 1 mM EDTA solution, pH 9.0) for 12 min at 98 °C in a microwave (TissueWave 2, Thermo Scientific). After cooling, the sections were rinsed with PBS (2 × 5 min) and 0.05% Tween-20 (822,184, Merck, Germany) in PBS (PBST) (5 min) and blocked 1 h with 1% bovine serum albumin (BSA) (A8022-100G, Life Technologies, USA) in PBST at RT in a humidified chamber. For the immunofluorescence labelling, primary antibodies diluted in blocking buffer were added, and the slides were incubated overnight at 4 °C. Subsequently, the slides were rinsed with PBS (2 × 5 min) and PBST (5 min) and incubated 1 h with the secondary antibodies diluted in blocking buffer at RT. Next, the slides were rinsed with PBS (2 × 2 min), PBST (2 min), distilled water (2 min) and mounted with Pro-Long Gold (P36930, Life Technologies, USA). Negative controls were obtained by omitting the primary antibodies. The primary antibodies used were goat anti-FOXL2 (1:200, NB100-1277, Bio-Techne), mouse anti-AMH (1:30, MCA2246, R&D system), rabbit anti-KRT19 (1:100, ab76539, Abcam), goat anti-DDX4 (1:250, AF2030, R&D), rabbit anti-KI67 (1:100, ab15580, Abcam), mouse anti-STAR (1:100, sc166821, Santa Cruz) and mouse anti-PCNA (1:100, sc-56, Bio-connect). The secondary antibodies used were Alexa Fluor 488 donkey anti-rabbit IgG (1:500, A21206, Life Technologies), Alexa Fluor 647 donkey anti-goat IgG (1:500, A-21447, Life Technologies) and Alexa Fluor 594 donkey anti-mouse IgG (1:500, A21203, Life Technologies). For the TUNEL-assay, the In Situ Cell Detection Kit FITC (11684817910, Roche, Germany) was employed following the manufacturer’s instructions. The nuclei were stained with 4′,6-diamidino-2-phenyl-indole (DAPI) (1:1000, D1306, Life Technologies, USA), and sections were mounted using Pro-Long Gold.

### Statistics

Data distribution was determined using histograms and the Shapiro–Wilk test. Normally distributed continuous variables are presented as mean ± SD and skewed data as median (interquartile range (IQR)). Categorical variables are presented as percentages. Statistical tests were performed with the total number of endometriomas. Correlations between follicle density and, in particular (small), antral follicle density, and patient, surgical- and endometrioma characteristics were assessed by linear regression analyses. All variables that associated with follicle density in univariable analyses (*p* < 0.10) were included in a multivariate analysis to adjust for potential confounding. Statistical tests were considered as significant if the two-tailed *p* value was < 0.05. A Bonferroni correction was used to correct for multiple testing. A nonparametric Mann–Whitney *U* test was performed to compare follicle density in the following subgroups; patients with deep endometriosis (DE), hormonal therapy users, previous ipsilateral ovarian cystectomy *versus* no ovarian surgery and patient’s age of tissue surrounding endometriomas wherein (small) antral follicles were found *versus* endometriomas where no (small) antral follicles were found. Pearson’s chi-squared test was performed to compare the difference in hormonal therapy usage between the 5-year age groups. All statistical tests were performed using IBM SPSS version 25.0 for Windows.

## Results

### Patients

A total of 337 patients were screened for inclusion after which 69 patients were enrolled as study participants. One patient was subsequently excluded upon thorough review, as the primary indication for surgery was septic shock caused by an ovarian abscess, which (in retrospect) turned out to be an infected endometrioma. A flow chart illustrating the patient inclusion process is presented in Supplementary Fig. 1. Baseline characteristics are outlined in Table 1. Among the participants, 20 (29.4%) used hormonal therapy at the time of surgery of which nine (45.0%) were on a combination therapy of oestrogen and progestin, five (25.0%) were using GnRH analogues and six (30.0%) were on progestin-only therapy. The indications for surgery were pain (44.1%) and fertility (23.5%) of a combination of these (26.5%) (Table 1).

**Table 1:** Baseline characteristics

Patient population (*n* = 68)		
Age (years), mean ± SD		33.43 ± 5.6
BMI (kg/m^2^), mean ± SD		24.0 ± 4.0
Parity, *n* (%)	0	58 (85.3%)
	1	6 (8.8%)
	≥ 2	4 (5.9%)
Wish to conceive in the future, *n* (%)		37 (54.4%)
Hormonal therapy, *n* (%)		20 (29.4%)
Previous ovarian cystectomy, *n* (%)		11 (16.2%)
	Index ovary	10 (14.7%)
Primary indication for surgery, *n* (%)	Pain	30 (44.1%)
	Fertility	16 (23.5%)
	Combination	18 (26.5%)
	Other	4 (5.9%)
Removed endometriomas per patient, median (IQR)		1.0 (1.0–2.0)
Diameter endometrioma in cm, median (IQR)		4.3 (3.0–6.0)
rASRM*, median (IQR)		4.0 (3.0–4.0)
AMH before surgery (µg/L)**, median (IQR)		2.15 (0.7–4.4)
Analysed pathology slides per endometrioma, median (IQR)		2.0 (2.0–3.0)
Analysed ovarian tissue volume per endometrioma in mm^3^, median (IQR)		1.7 (1.0–2.6)

*BMI*, body mass index; *AMH*, anti-Müllerian hormone; *rASRM*, revised American Society for Reproductive Medicine score; *IQR*, interquartile range.*missing data *n* = 19.**missing data *n* = 39

### Histologic analysis

Pathology slides of 96 endometriomas removed from 68 patients were analysed. Among these patients, 44 had unilateral endometrioma(s), 23 had bilateral endometriomas and one patient presented with three endometriomas. A median of two slides per endometrioma were available for analysis, which corresponded to a median of 1.7 mm^3^ of ovarian tissue. Ovarian tissue attached to the cyst wall was observed in 90 out of 96 endometriomas (93.7%) and from those 81 contained follicles (84.4%). In the other six endometriomas, only cysts wall or scar tissue was observed (6.3%).

In total, 1944 follicles were classified according to maturation stage. An overview of follicle distribution across 5-year age groups is shown in Table 2. A median of 2.99 follicles/mm^3^ (IQR 0.61–11.00) was found. The highest follicle density was observed in ovarian cortex tissue of patients aged between 21 and 25. The mean follicle diameter per maturation stage is depicted in Supplementary Table 1.

**Table 2:** Distribution of follicles across 5-year age groups in ovarian tissue attached to stripped endometriomas

Age groups	Primordial *n* (%)	Intermediate primordial *n* (%)	Primary *n* (%)	Intermediate primary *n* (%)	Late primary *n* (%)	Small antral *n* (%)	Antral *n* (%)	Total *n* (%)	Follicle density* median (IQR)
21–25 (*n* = 9)	211 (41.1%)	199 (38.7%)	30 (5.8%)	34 (6.6%)	13 (2.5%)	3 (0.6%)	24 (4.7%)	514 (100%)	18.81 (5.92–36.21)
26–30 (*n* = 25)	313 (33.1%)	313 (33.1%)	157 (16.6%)	86 (9.1%)	38 (4.0%)	5 (0.5%)	35 (3.7%)	947 (100%)	9.29 (2.56–20.57)
31–35 (*n* = 29)	69 (30.1%)	77 (33.6%)	37 (16.2%)	19 (8.3%)	11 (4.8%)	1 (0.5%)	15 (6.6%)	229 (100%)	2.92 (1.03–5.63)
36–40 (*n* = 25)	73 (43.7%)	42 (25.2%)	28 (16.8%)	5 (3.0%)	5 (3.0%)	0 (0.0%)	14 (8.4%)	167 (100%)	0.42 (0.0–2.35)
41–46 (*n* = 8)	27 (31.0%)	25 (28.7%)	18 (20.7%)	10 (11.5%)	4 (4.6%)	0 (0.0%)	3 (3.5%)	87 (100%)	2.58 (0.70–14.90)
Total	693 (35.7%)	656 (33.7%)	270 (13.9%)	154 (7.9%)	71 (3.7%)	9 (0.5%)	91 (4.7%)	1944 (100%)	2.99 (0.61–11.00)

*IQR*, interquartile range. *Follicles/mm^3^

In total, nine small antral and 91 antral follicles were identified in ovarian cortex tissue surrounding 35 endometriomas (36.5%) (Table 2). The median follicle density of (small) antral follicles in this group was 0.91 follicles/mm^3^ (IQR 0.48–1.5). Comparing the group of endometriomas with (small) antral follicles to the group without, a significant difference in the median age was observed (30 *versus* 35 years, *p* = 0.003).

### Patient and surgical characteristics in relation to follicle density

Next, we assessed the median follicle density in subgroups according to presence of deep endometriosis, use of hormonal therapy and previous ipsilateral ovarian cystectomy. The surgical #Enzian classification was only available from women who underwent surgery in the HMC. This subgroup analysis revealed no significant difference in follicle density between the selected subgroups (Supplementary Table 2).

To correct for potential confounders, we conducted both univariate and multivariate linear regression analysis. Table 3 demonstrates a significant negative correlation between follicle density and age. Although not statistically significant after application of the Bonferonni correction, a negative trend between endometrioma diameter and follicle density was observed in the multivariate analysis.

**Table 3:** Linear regression analysis for follicle density

	*n*	Univariable		Multivariable	
		*B*^a^	*p* value	*B*^a^	*p* value
Age per 1-year increase	96	–1.217	< 0.001	–1.265	< 0.001
BMI per 1 kg/m^2^ increase	96	0.494	0.276		
Diameter endometrioma per 1-cm increase	96	–1.363	0.098	–1.594	0.038
Previous ipsilateral ovarian cystectomy^b^	12	–6.626	0.240		
rASRM per 1 point increase	70	1.106	0.761		
AMH before surgery per 1 µg/L increase	29	0.332	0.837		

*AMH*, anti-Müllerian hormone; *rASRM*, revised American Society for Reproductive Medicine score; *BMI*, body mass index. ^a^Unstandardized regression coefficient. ^b^Reference group: previous contralateral cystectomy or no previous ovarian cystectomy

Additionally, we performed a linear regression analysis to identify patient or surgical characteristics that may influence the density of (small) antral follicles (Supplementary Table 3). A significant negative correlation between age and (small) antral follicle density (*p* = 0.001) was found. After adjustig for age, BMI was not significantly associated with (small) antral follicle density in the multivariate regression analysis.

### Immunofluorescence

**Fig. 2:**
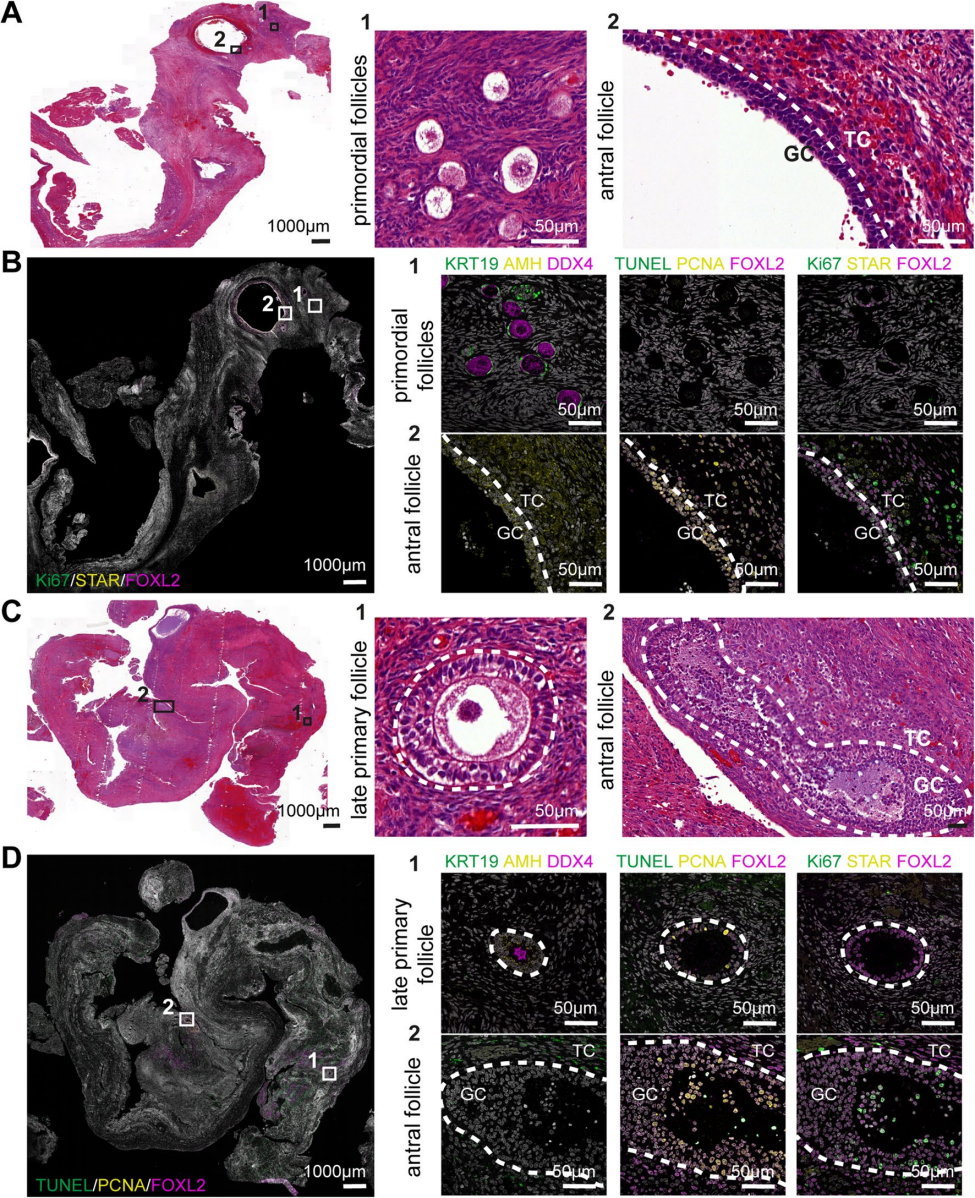
Characterisation of follicles in ovarian cortex tissue attached to stripped endometriomas. **A** H&E slide of ovarian tissue attached to stripped endometrioma tissue derived from patient 1 showing antral follicles. (A1) Healthy primordial follicle pattern. (A2) Antral follicle wall showing organised granulosa cell (GC) and theca cell (TC) structure. **B** Immunofluorescence for Ki67, STAR and FOXL2. (B1) Primordial follicles showing DDX4, KRT19, FOXL2 and Ki67 expression. (B2) Antral follicle wall showing AMH, FOXL2, PCNA, Ki67 and STAR expression. **C** H&E slide of ovarian tissue attached to stripped endometrioma tissue derived from patient 2 showing (small) antral follicles. (C1) H&E staining of a late primary follicle. (C2) H&E staining of an antral follicle showing disorganised GC and TC structure. **D** Immunofluorescence for KRT19, AMH and DDX. (D1) Late primary follicle showing DDX, AMH, FOXL2, PCNA and Ki67 expression. (D2) Antral follicle showing KRT19, FOXL2, PCNA, Ki67 and little TUNEL expression

To assess the quality of the (small) antral follicles, immunofluorescent staining was performed on a subset of ovarian cortex tissue attached to endometriomas. We selected four endometriomas derived from four patients, where antral follicles were observed in the H&E slides (Fig. 2). The patients’ ages and corresponding endometrioma diameters were as follows: patient 1: 24 years, 6 cm; patient 2: 32 years, 4.6 cm; patient 3: 37 years, 4 cm; patient 4: 27 years, 7 cm.

In two out of the four endometriomas (patients 1 and 3), healthy antral follicles could be observed. Many follicles showed a regular structure and normal organisation of granulosa cells (KRT19 + cells in primordial follicles and AMH + /FOXL2 + in growing follicles) and theca cells, positive signal for proliferation makers (Ki67, PCNA), and no evidence of late apoptosis was detected (TUNEL) (Fig. 2B, patient 1). The single channel images are included in Supplementary Fig. 2. Some theca cells in the antral follicles expressed no/low levels of STAR, suggesting that they are non-steroidogenic, and several antral follicles contained many granulosa cells in the antral cavity and rounded theca cells, potentially suggesting early atresia (Fig. 2D, patient 2). The single channel images are included in Supplementary Fig. 3.

## Discussion

This study aimed to assess the follicle density in ovarian cortex tissue attached to stripped endometriomas, with a focus on (small) antral follicle density. Our data demonstrates the existence of follicles with a median density of 3 follicles/mm^3^ in ovarian tissue adjacent to removed endometriomas. Follicle density was negatively affected by the age of the patient. Antral follicles (< 7 mm) were present in 36.5% of cases and comprised a density of 1 follicle/mm^3^, which was negatively affected by the age of the patient. Particularly in patients aged 30 or younger, antral follicles were present. With regard to the quality, based on immunofluorescence, healthy antral follicles were present in this tissue (2 out of 4 examined cases). From this study, we conclude that there is potential (based on antral folilcle density) to conduct future research to explore the application of IVM on antral follicles isolated from ovarian tissue attached to stripped endometriomas. This is the first study reporting on antral follicle density in this tissue. In addition, we are the first to introduce the concept of exploring whether IVM could serve as a novel approach for preserving oocytes from ovarian tissue that is inadvertently removed during endometrioma cystectomy.

The stripping technique is associated with unintentional removal of ovarian tissue alongside the ovarian cyst wall, especially in cases of endometriomas [23, 28]. Even when performed by highly experienced surgeons (as in our centres), the ovarian reserve will be reduced after surgery [6]. However, the stripping technique is often the preferred choice of treatment due to the higher reported (spontaneous) pregnancy rates and the lower chance of recurrence compared to other surgical techniques, such as laser vaporisation [29, 30]. In our study, we observed ovarian tissue attached to 93.7% of endometriomas, which is in agreement with percentages found in other studies [8, 11, 12]. On the contrary, some report lower percentages ranging from 53 to 65%. This discrepancy could be attributed to lower inclusion numbers, cyst location (surface or deep within the ovary) and to co-existence of deep endometriosis, which has been identified as a risk factor for removal of ovarian tissue during cystectomy [9, 10, 23].

Previous research has evaluated total follicle density in this tissue compared to other benign lesions. Schubert, et al. reported a total follicle density of 0.31 follicles/mm^3^, which was lower compared to the density found in ovarian tissue attached to dermoid and serous cysts [2]. In contrast, our data demonstrates a notably higher follicle density of 2.99 follicles/mm^3^. This could be potentially explained by the lower inclusion numbers (*n* = 13) and the inclusion of larger endometriomas (mean 56.9 mm ± 5.4) in the study of Schubert, et al*.*, as opposed to the current study. When comparing our findings to the follicle density in healthy ovarian tissue (16.3 follicles/mm^3^, *n* = 100), we observed a significantly lower follicle density [31]. These results are consistent with the findings from another study that compared follicle density in ovarian cortex tissue biopsies from healthy ovaries to tissue from ovaries affected by endometriomas [32].

The current study supports the age-dependent decline in follicle density, which has been well established in literature [22]. With regard to endometrioma size, our results, based on 96 included endometriomas, are in contrast with the results of Schubert, et al. (*n* = 13), and show a negative trend (*p* = 0.038) between endometrioma volume and follicle density [2]. From our data, it can be speculated that the mechanically, space-occupying effect of the endometrioma contributes to the decrease in follicle density. This is supported by a recent study that demonstrated a significant negative impact of endometrioma volume on AMH levels [33]. No correlation between pre-operative AMH levels and follicle density was observed in our study, but this could be the result of incomplete data. However, other studies also showed poor predictive value of serum AMH for ovarian follicle density [34].

Our observations revealed the presence of healthy follicular patterns in some of the tissues. This contradicts the assertion made by Muzii et al. who concluded the absence of viable follicular patterns, though they only included 26 endometriomas [23]. Furthermore, 5% of the total follicles contained a (small) antrum, which is consistent with previously reported results [18].

Immunofluorescence showed no signs of late apoptosis (TUNEL) in some antral follicles, yet the low levels of STAR expression may suggest the potential initiation early apoptosis. However, the oocyte itself may not be affected by this process, as previously described [35].

Strengths of our study include a substantial sample size of endometriomas in contrast to other studies in this field, which enabled us to perform additional analysis and to identify variables of predictive value for future IVM studies [2, 18, 19, 23, 32]. In addition, we provide the follicle density of (small) antral follicles in particular.

Our study, however, is limited. The retrospective study design may have induced confounding and information- and selection bias. We minimised this level of bias by multiple regression analysis, clear selection of the study population prior to histological analysis, blinding of patient information during the analysis phase and the usage of a standard analysis method for follicle classification. Tissue analysis was constrained by the number of available pathology slides per patient, not fully representing the follicle distribution in the removed ovarian tissue, nor the whole ovary. Indeed, previous research has demonstrated the heterogenic distributions of follicles in healthy ovarian cortex tissue [36]. This could explain the unexpectedly low median follicle density and relatively high number of antral follicles in age group 36–40. In addition, the heterogeneous distribution of follicles may also explain why we observed nine small antral follicles *versus* 91 antral follicles. According to the follicular growth model of Gougeon, it takes ~ 60 days for a small antral follicle (0,4-mm diameter) and ~ 20 days for an antral follicle (2-mm diameter) to reach the ovulatory stage [27]. Hence, one would expect to find more small antral follicles compared to antral follicles. Furthermore, potential observer bias might have resulted from the assessment of pathology slides by a single observer. To mitigate this, all follicles with unclear developmental stages were discussed with a second observer to ensure data integrity. Furthermore, information about the menstrual cycle at the time of surgery (known to influence follicular density) was not reported [33]. Nevertheless, we did not observe a significant difference in follicular maturation stage between women using hormonal therapy at the time of surgery *versus* women who did not (data not shown). To conclude, we acknowledge that the immunofluorescent analysis was conducted on paraffin-embedded tissue derived from a small number of endometriomas, potentially limiting the generalisability of our findings. However, the goal of this study was to determine whether healthy antral follicles (based on immunofluorescence) are indeed present next to the atretic ones (a normal physiological phenomenon in the ovary) [27], before proceeding to research using fresh tissue. Because of these limitations, further research is necessary to provide more data on the viability of the follicles to conclude that they are feasible to use for IVM.

Nikiforov et al. examined the maturation of 895 immature oocytes (with a similar diameter compared to the (small) antral follicles in the current study) derived from cancer patients with an indication for OTC. They reported a metaphase II maturation rate of one in three (Supplementary Table 2) [20]. However, there are concerns regarding follicle quality in the tissue examined in this study [24]. Immunofluorescent analysis indicated the presence of healthy antral follicles in two out of four cases. We believe that our findings encourage to continue research with fresh tissue. Therefore, at the LUMC, a centre with extensive experience with the IVM technique, we are currently setting up a study to isolate (small) antral follicles from ovarian tissue attached to freshly stripped endometriomas for subsequent IVM procedures.

In conclusion, in the majority of cases, healthy ovarian tissue is removed during endometrioma cystectomy. This situation calls for innovating approaches on either how to prevent or minimise this or on how to transform the collateral surgical damage to benefit our patients, rather than discarding it. Our study suggests that ovarian tissue attached to stripped endometriomas represents a potential and non-invasive source for (small) antral follicles. Application of IVM could be theoretically an interesting alternative fertility preservation option in young patients with endometriomas who are undergoing surgery, but more research assessing the viability of these follicles in fresh tissue is necessary.

### Supplementary Information

Below is the link to the electronic supplementary material.

Supplementary file1 (DOCX 107 KB)

Supplementary file2 (TIF 17866 KB)

Supplementary file3 (TIF 19696 KB)

Supplementary file4 (DOCX 22.6 KB)
