# Supplementary figures and images for: Histological analysis of (antral) follicle density in ovarian cortex tissue attached to stripped endometriomas
Source: J Assist Reprod Genet. 2024 Mar 5;41(4):1067–76. doi: 10.1007/s10815-024-03058-0 (PMC11052973; doi:10.1007/s10815-024-03058-0)

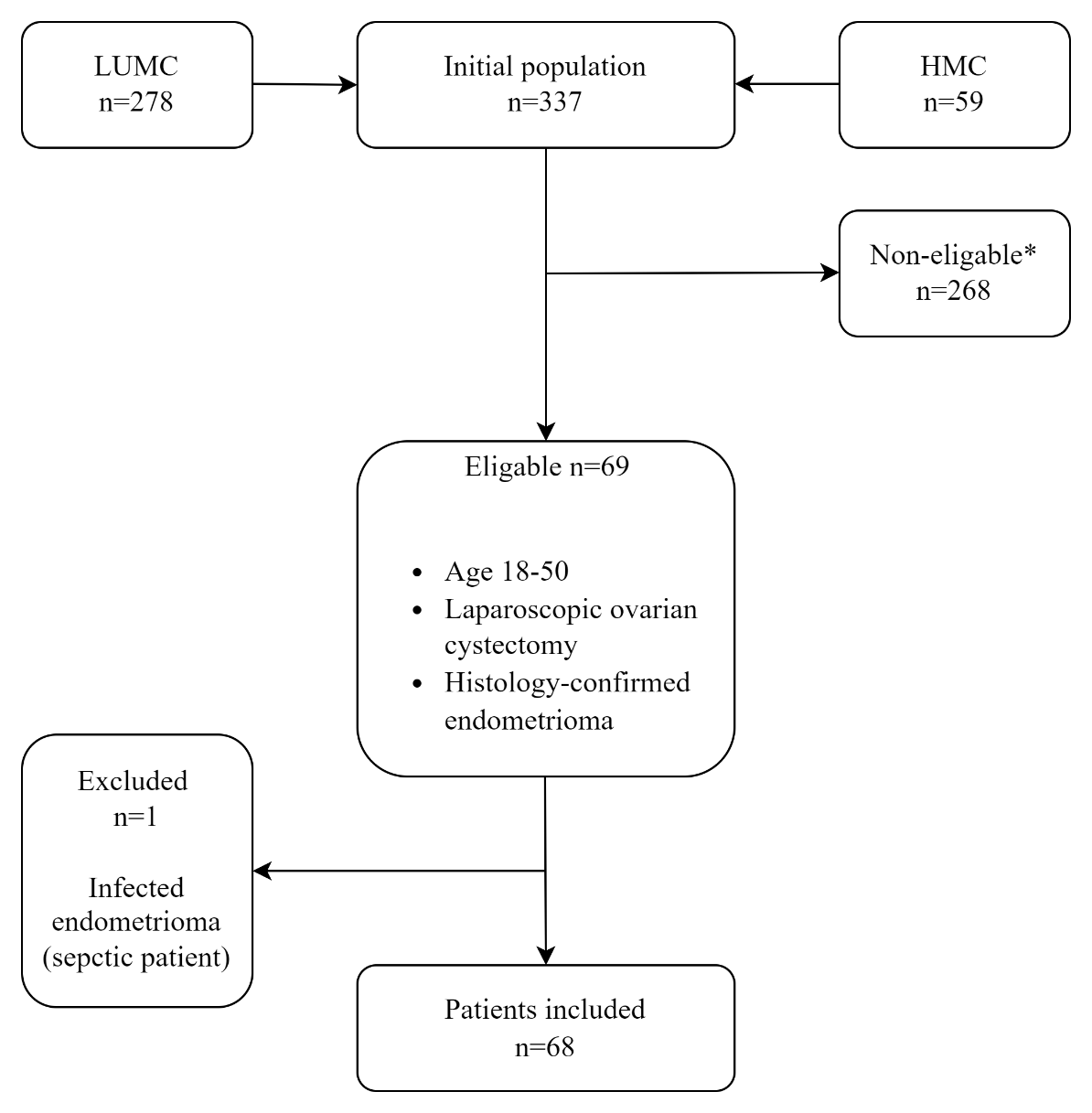

Supplement: Supplementary file 1 — Supplementary file1 (DOCX 107 KB) [file 10815_2024_3058_MOESM1_ESM.docx]

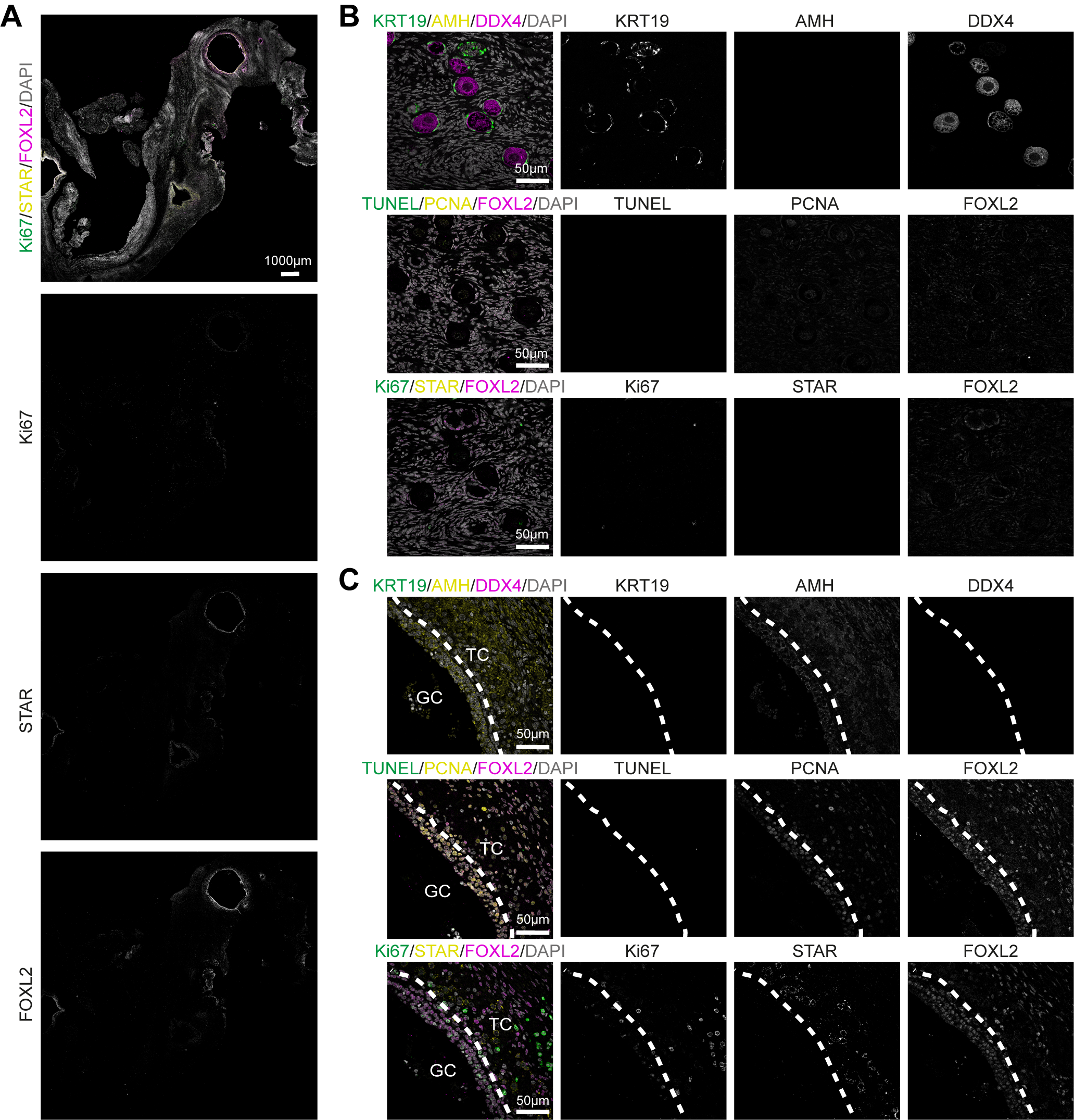

Supplement: Supplementary file 2 — Supplementary file2 (TIF 17866 KB) [file 10815_2024_3058_MOESM2_ESM.tif]

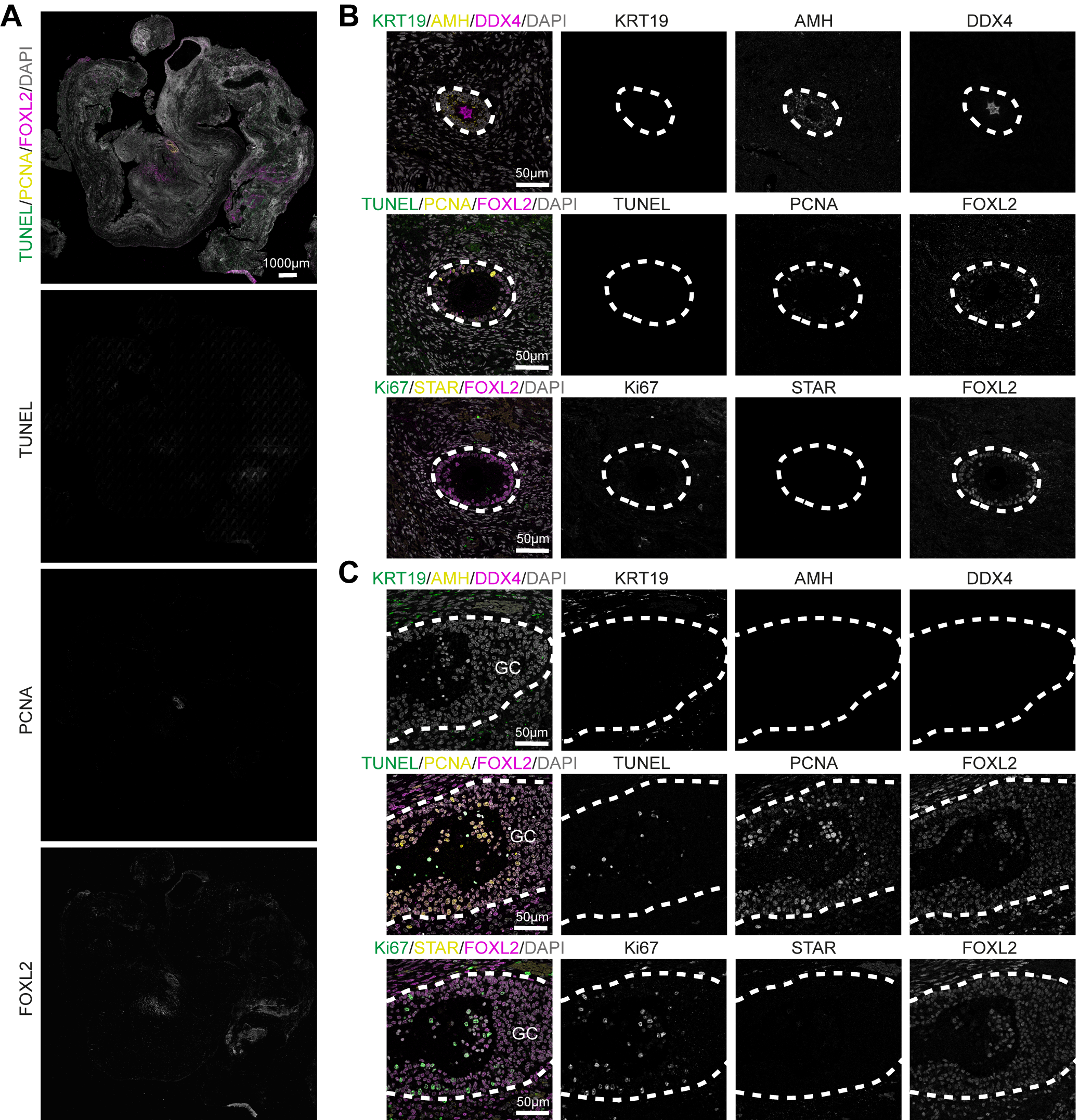

Supplement: Supplementary file 3 — Supplementary file3 (TIF 19696 KB) [file 10815_2024_3058_MOESM3_ESM.tif]
