# Supplementary material for: Histological analysis of (antral) follicle density in ovarian cortex tissue attached to stripped endometriomas
Source: J Assist Reprod Genet. 2024 Mar 5;41(4):1067–76. doi: 10.1007/s10815-024-03058-0 (PMC11052973; doi:10.1007/s10815-024-03058-0)
Supplement: Supplementary file 4 — Supplementary file4 (DOCX 22.6 KB) [file 10815_2024_3058_MOESM4_ESM.docx]

**Supplementary Table 1. Follicle developmental stage and diameter**

| Follicle stage | n | Diameter (µm), mean±SD |
| --- | --- | --- |
| Primordial | 693 | 50.31±3.54 |
| Intermediate primordial | 656 | 55.92±3.93 |
| Primary | 270 | 65.96±11.25 |
| Intermediate primary | 154 | 85.89±10.20 |
| Late primary | 71 | 134.98±59.22 |
| Small antral | 9 | 270.19±125,16 |
| Antral | 91 | 1822,53±1393,57  <500, n=19  501-1000, n=13  1001-2000, n=24  2001-3000, n=17  3001-4000, n=10  4001-5000, n=5  5001-6000, n=2  6001-7000, n=1 |

**Supplementary Table 2. Subgroup analysis in relation to follicle density**

| Total number endometriomas (n=96) |  | n | Follicle density*  Median (IQR) | P value |
| --- | --- | --- | --- | --- |
| Deep endometriosis^a^ | yes | 41 | 3.16 (0.98-12.53) | 0.188 |
|  | no | 10 | 9.71 (2.98-18.32) |  |
| Hormonal therapy | yes | 26 | 3.81 (1.62-10.08) | 0.594 |
|  | no | 70 | 2.69 (0.59-11.35) |  |
| Previous ipsilateral ovarian cystectomy | yes | 12 | 2.08 (1.08-6.39) | 0.542 |
|  | no | 84 | 3.38 (0.59-11.96) |  |

IQR, interquartile range; *Follicles/mm^3; a^ Presence of deep endometriosis in #Enzian compartment A, B, C or F during surgery, missing values: n=45 (not included in analysis).

**Supplementary Table 3. Linear regression analysis for (small) antral follicle density**

|  | n | Univariable | | Multivariable | |
| --- | --- | --- | --- | --- | --- |
|  |  | B^a^ | P value | B^a^ | P value |
| Age per 1 year increase | 96 | -0.062 | 0.001 | -0.060 | 0.001 |
| BMI  per 1 kg/m^2^ increase | 96 | 0.045 | 0.071 | 0.040 | 0.088 |
| Diameter endometrioma per 1 cm increase | 96 | -0.022 | 0.630 |  |  |
| Previous ipsilateral ovarian cystectomy^b^ | 12 | -0.025 | 0.935 |  |  |
| rASRM  per 1 point increase | 70 | -0.230 | 0.262 |  | |
| AMH before surgery per 1 µg/L increase | 29 | 0.112 | 0.186 |  | |

AMH, anti-Müllerian hormone; BMI, body mass index; rASRM, revised American Society for Reproductive Medicine score; ^a^ Unstandardized regression coefficient. ^b^ Reference group: previous contralateral cystectomy or no previous ovarian cystectomy.
